# The Impact of Artificial Intelligence on Dental Implantology: A Narrative Review

**DOI:** 10.7759/cureus.47941

**Published:** 2023-10-30

**Authors:** Abdulaziz M Altalhi, Faisal S Alharbi, Mazen A Alhodaithy, Bandary S Almarshedy, Majida Y Al-saaib, Raneem M Al jfshar, Abdulrahman S Aljohani, Adeeb H Alshareef, Mona Muhayya, Noura H AL-harbi

**Affiliations:** 1 Dentistry, Ministry of Health, Riyadh, SAU; 2 Family Medicine, Dentistry, Ministry of Health, Riyadh, SAU; 3 Dentistry, King Saud University, Riyadh, SAU; 4 Dentistry, Safwat Al Muhaidib Dental Group, Yanbu, SAU; 5 Dentistry, King Saud Bin Abdulaziz University for Health Sciences, Riyadh, SAU

**Keywords:** artifical intelligence and robotics, artifical intelligence, robot-assisted, ai, dental implant

## Abstract

Implant dentistry has witnessed a transformative shift with the integration of artificial intelligence (AI) technologies. This article explores the role of AI in implant dentistry, emphasizing its impact on diagnostics, treatment planning, and patient outcomes. AI-driven image analysis and deep learning algorithms enhance the precision of implant placement, reducing risks and optimizing aesthetics. Moreover, AI-driven data analytics provide valuable insights into patient-specific treatment strategies, improving overall success rates. As AI continues to evolve, it promises to reshape the landscape of implant dentistry and lead in an era of personalized and efficient oral healthcare.

## Introduction and background

Artificial intelligence (AI), a branch of computer science, is a fast-growing field in healthcare. This term was first introduced in 1956 at Dartmouth University [1]. By definition, it is “the theory and development of computer systems able to perform tasks normally requiring human intelligence, such as visual perception, speech recognition, decision making, and translation between languages” [2]. The major subfields of AI include machine learning (ML), deep learning (DL), artificial neural networks (ANNs), and robotics [3]. In dentistry, ML and its subset DL have found a major role in diagnosis, decision-making, predicting treatment outcomes, and treatment planning.

ANNs consist of an input layer, an output layer, and multiple small communicating units called neurons, which are organized in layers. Convolutional neural networks (CNNs), a subset of an ANN, are commonly used in medicine and dentistry. By using a mathematical operation called convolution and a special neuron architecture, a CNN processes the input data [4]. In dentistry, the input data can mainly be in the form of text (case records, laboratory reports), images (clinical images, x-rays), or sounds (handpiece sounds). A CNN uses filters to scan small amounts of data at a time. These filters, also known as kernels, help the network detect patterns and features at different spatial scales. The pooling layers then reduce the complexity of the network, making it stronger to the variations in the object position and scale [5]. AI generally operates in two phases - the training phase and the testing phase. In the training phase, the AI model learns the features, relationships, and patterns of the data. The goal of this phase is to teach the model to make accurate predictions or decisions. In the testing phase, the trained AI model gives predictions or decisions for new data that it has not encountered during the training phase [6].

Implant dentistry has advanced exponentially all over the globe over the last few decades. Implantology has changed the face of dentistry, especially for the rehabilitation of patients with single, partial, or complete edentulism. There have been enough literature reports on the improvements in the patient’s overall quality of life following implant treatment [7-8]. Continued research and advanced technologies have improved the success and survival rates of implants. The advancements in this field have, however, come with their share of complications, such as peri-implantitis, prosthetic complications, and issues with super-structures or the implant. Managing implant complications is a challenging task, as highlighted by Hanif et al. [9].

This narrative analysis examines the prevailing literature, emphasizing the integration and implications of AI in dental implantology. A pivotal inquiry guiding our review is "In what ways is AI influencing dental implantology across areas such as treatment planning, implant brand distinction, design innovation, outcome prediction, and robotic-assisted surgeries?" Through this detailed exploration, we underscore AI's increasing significance in refining the domain of implant dentistry.

## Review

Search methodology

We conducted a comprehensive search using databases such as PubMed, Google Scholar, Embase, and MEDLINE, utilizing the keywords "Implant AND AI" and "Implant AND Artificial Intelligence." Our focus was on extracting all relevant clinical trials, systematic reviews, and meta-analyses. We included studies exclusively published in English. Initially, we excluded articles based on irrelevance in their titles. Subsequently, a thorough review of abstracts was undertaken, which further narrowed down the selection to the most pertinent studies concerning our subject matter.

Applications of AI to implant dentistry

Assist in Treatment Planning

Cone beam CT (CBCT) scans are the gold standard for dental implant planning all over the world. General dental practitioners may not have the necessary skill sets to evaluate CBCT scans for detailed implant planning and identification of anatomical structures. AI can contribute to solving this problem. Bayrakdar et al. used DL for dental implant planning in CBCT images and noted limited success with the same [10]. They suggested that more extensive studies are required to train the AI model for bone height and thickness measurements. Moufti et al. compared the segmentation done by an AI model and a human investigator for a tooth-bounded mandibular edentulous area and noted the acceptable accuracy of the AI model [11]. This is the first stage of implant planning, and automation of bone-level assessment on a CBCT has further potential to reduce the overall time and cost required for dental implant treatment. Similar results were also obtained by Fontenele et al. for alveolar bone segmentation of the maxillary alveolar region [12]. However, they noted that the manual segmentation had a slightly better accuracy rate and the time required for the AI model was 116 times less than the manual approach.

While the measurement of bone height and width for implant placement by AI has met with limited success, research states that AI can serve as a valuable tool for the detection of anatomical landmarks. Kwak et al. noted the successful detection of the mandibular canal using a deep CNN model [13]. They stated that AI can serve as a reliable tool for canal determination and play a significant role in implant planning in the future. Similar results were also noted by Oliveira-Santos et al. where the mandibular canal, along with its variation (anterior loop), was determined by AI with high accuracy [14]. For the segmentation of the maxillary sinus, the AI model used by Morgan et al. provided consistent automatic segmentation, which could allow for the precise reproduction of 3D models for diagnosis and treatment planning [15].

A case report was published by Mangano et al. where they combined AI and augmented reality for guided implant surgery planning in a partially edentulous patient [16]. They believed that their novel protocol was efficient and time-saving for simple cases of guided implant surgery. An interesting study was performed by Sakai et al. where they used pre-op CBCT scans to predict implant drilling protocols [17]. Three drilling protocols were analyzed - conventional drilling protocol with a tapping drill, conventional drilling protocol without a tapping drill, and undersized drilling protocol. A precision accuracy of 93.7% was noted, thereby suggesting that AI can be used to predict the primary stability of implants based on the drilling protocols pre-surgery. This could be of great help to young clinicians who are at the start of their implantology careers.

Prosthetically driven implantology requires a precise 3D placement of the dental implant. As of today, the use of AI can be an asset to treatment planning in implantology by assisting clinicians in the decision-making process. Further research is required to let the AI model run point on 3D planning of the future dental implant.

Detection/Recognizing Implant Type/Brand

There are several brands of implants available currently all over the world. These implants have different abutments and different prosthetic components. In case of any complications with the implants or their super-structures, additional prosthetic, surgical, or periodontal procedures are required. Additional information, such as implant manufacturer, diameter, length, platform, and abutment type, is required during these problem-solving appointments. This information is easily accessible if the implant treatment was performed by the same clinician. If the procedures have been performed at another clinic, and the treatment provider cannot be contacted, it may be difficult or even impossible to get this information. The use of AI for implant brand detection is a potential solution to this increasingly complex problem.

In clinical practice currently, there are already two systems for implant detection. The first system uses a website (www.whatimplantisthat.com), which contains a database of radiographs of different implant brands wherein dentists are required to check if their radiograph images match the website image [18]. The second system developed by Michelinakis et al. uses a questionnaire about implant characteristics, and it requires the dentists to match the answers with the database to identify the implant [19]. However, both these systems require the clinician to match the radiographic image to the database, thus increasing the element of human error in the identification process. The advantage of AI is that the computer identifies the implant instead of the dentist. The CNNs of the DL family can identify images by forming an identification algorithm in which they can detect the spatial hierarchies of features, such as edges, textures, and shapes [20].

Literature on the accuracy and feasibility of the detection of different dental implant systems (DIS) by AI is now emerging. A systematic review by Chaurasia et al. concluded that DL should be used as a decision-aid tool for experienced clinicians to increase the accuracy of the detection of DIS [21]. They believed that, since DL algorithms are constantly evolving, it is not possible to classify DIS solely based on these data, and clinical knowledge should be backed by AI to make this decision. In a pilot study by Takahasi et al., 1,282 panoramic radiographs with six implant systems from three manufacturers were used as a dataset [22]. Specifically, 80% of the images were used as a training dataset, and 20% as the testing dataset. The mean average precision of their model was 0.71, and the mean intersection over union was 0.72. A systematic review by Revilla-León et al. noted the overall accuracy range of 93.8%-98% of the AI models in the different reviewed studies [23]. Most of the reviewed studies extracted data from a 2D radiograph, such as a periapical or panoramic radiograph, instead of CBCT. As of now, CBCT has not been used for data extraction to train AI models. This is also supported by a study by Correa et al. who have raised queries on the resolution and sharpness of CBCT and suggested that it may be lower as compared to peripheral radiographs [24]. Hence, whether CBCT can be used for the classification of DIS by AI is debatable [24].

There have been multiple studies with a diverse number of DIS and variable datasets to test the accuracy of AI in image detection. A multi-center study evaluating 156,965 panoramic and periapical radiographs by Park et al. noted high accuracy for both 2D radiographs [25]. Similar results were obtained in several other studies [20,26-28]. All these studies, while noting the high accuracy of the AI model in the identification of DIS, have suggested expanding the dataset to incorporate more images of implant brands to get more precise results. The results of all these studies, however, cannot be co-related as they are all performed under different conditions, with different CNN models, varied numbers of training and test images, and different implant brands.

The major implant systems vary in different parts of the world. Creating an accurate database for each implant is the need of the hour. The formation of a regional-based DL model for accuracy verification is also very important. Adherence to medical ethics while using big data, for building a global dental implant classification system, will effectively contribute to dental care all over the world.

Development of New Implant Designs

There have been a few studies that have applied an AI model for implant design optimization using finite element analysis (FEA). FEA is a mathematical model that determines the mechanical behavior of dental implants, especially stress concentration at the implant-bone interface [29]. Li et al. developed an AI model to measure the stress at the implant-bone interface by considering the implant length, implant thread length, and thread pitch [30]. This model, in comparison with the FEA model, noted a reduction of 36.6% stress at the interface. Roy et al. proposed to modify the implant geometry (implant length, porosity, and diameter) with a combination of ANNs and genetic algorithms to achieve the desired micro-strain at the implant-bone junction [31]. Zaw et al. used a reduced-basis method to train a neural network model to accurately measure the elastic modulus of the bone-implant interface [32]. Further research is required to improve the applicability of AI in the development of new implant designs with more in vitro, animal, and clinical studies.

Prediction of Treatment Outcomes in Implantology

As dental implants continue to be the most preferred treatment modality for both patients and clinicians, implant complications are also on the rise. Implant complications lead to increasing costs and additional procedures for both the patient and the clinician. It is difficult to predict implant loss or its complications since there are many risk factors involved, such as patient characteristics, type and quality of alveolar bone, implant type, and surgical plan. Implant failure or loss is generally predicted by clinicians based on their clinical knowledge and experience. Prediction of treatment outcomes in implantology is the need of the hour, and AI has the potential to be a major contributor to this field.

The literature in this field is currently very limited with only singular articles with very limited follow-up. No systematic reviews and meta-analyses have yet been published on the prediction of treatment outcomes in implantology. Lyakhov et al. proposed a neural network model for predicting survival rates of single dental implants by analyzing the statistical factors of the patients [33]. They formulated their database based on the case histories and the clinical condition of the patient. Their model noted an accuracy rate of 94.48% for single implant survival. They, however, concluded that this model cannot be independently used for decision-making but can surely assist the clinician as a diagnostic tool in implantology.

Oh et al. noted that osteointegration of dental implants can be predicted to some extent by AI with plain radiographs and can complement the existing osseointegration determination methods [34]. Seven different DL models compared two groups of implants - one which was immediately placed and the others were radiographed after successful osteointegration. Cha et al. used an ML model to measure peri-implant bone loss on periapical radiographs [35]. While they believed that the model could assist clinicians in diagnosing and classifying peri-implantitis, in the current study, they did not find any statistically significant difference between the bone loss levels calculated by the dentists and the AI model.

Literature has reported articles that have predicted the risk of implant loss using neural networks. Huang et al. suggested that their predictive AI model can suggest the implant fate within five years, which will help dentists identify high-risk patients and accordingly modify their treatment plans [36]. Three models - a clinical model, a DL-based radiographic model, and an integrated model (by combining the clinical and radiological DL model) - were developed to predict the five-year implant loss risk. The integrated model had the best prediction rate as far as five-year implant loss risk was concerned. Another DL model predicting implant loss was developed by Zhang et al. based on periapical and panoramic films [37]. Per-implant alveolar bone loss levels were analyzed, and a prediction accuracy of 87% was obtained. The results of this study were also in accordance with the results of Huang et al. [36], where the model can accurately predict the risk of implant loss, thus aiding clinicians in the decision-making process. Further clinical evidence with long-term studies with a greater number of implants and brands is required before fully integrating AI into clinical practice.

Robotic Implant Surgery

The integration of robotics and AI in dentistry is called "dentronics" [38]. Precise surgical placement of a dental implant is essential to prevent any complications in both the surgical and prosthetic phases. In 2017, the Food and Drug Administration (FDA) approved the robotic surgical assistant for the placement of dental implants. Based on CBCT scans, the implant position is planned by the dentist, and the robotic arm performs the surgery with the dentist observing the procedure in real time - which gives the dentist the flexibility to change any angulations intraoperatively [38]. Such a case was performed in China in 2017 where two implants were placed in a patient by a robot without any intervention by the dentist. Several clinical reports have been published in the literature where successful implant placements have been done by robots [39-41].

A probable reason for robotics being a low-demand field in dentistry is the lack of expert knowledge. Additionally, research in this field requires collaboration between dentists and engineers. The use of AI can help the emergence of robotics in implant dentistry. AI can analyze large patient datasets to help in diagnosis and treatment planning, thereby optimizing the implant process.

Further scope

While applications of AI in implant prosthodontics have not yet been published extensively, a retrospective clinical study by Lerner et al. demonstrated the use of AI in the restoration of 90 patients with 106 implant-supported monolithic zirconia crowns [42]. They utilized the AI feature of computer-aided design (CAD) software to fabricate the final restoration that confirms the gingival contour even after tissue maturation following a temporization phase. Even though the AI used in this method was a "weak" AI, they believed that this method would allow dental technicians to save time and reduce the costs and errors of the final prosthetic process.

A study by Hwang et al. developed a DL model for automatic classification of surgical plans for sinus augmentation procedures in the maxillary posterior region [43]. They utilized the anatomical landmarks noted on a CBCT scan to train their AI model. The classification put forth by their model was then compared to the ABC classification proposed by Wang et al. [44]. Accurate detection of the anatomical landmarks and accurate classification of the sinus floor augmentation procedures make it a handy tool while planning the rehabilitation of patients with missing maxillary posterior teeth.

## Conclusions

In the dynamic arena of dental implantology, the fusion of AI emerges as a seminal force poised to redefine the landscape. This narrative review embarked on a systematic exploration, dissecting the multifaceted roles of AI, each contributing distinctively to the field. AI's influence is unmistakable, enhancing treatment planning precision, enabling implant brand differentiation through ML, fostering innovative implant designs with finite element analysis, and even venturing into outcome prediction and the promising realm of robotic-guided implant surgeries. Nonetheless, it is vital to recognize inherent limitations. The review, while comprehensive in breadth, prioritized an overarching perspective over an exhaustive examination of individual facets. The objective was to paint a panoramic view of AI's myriad contributions to implant dentistry.

In summary, AI's transformative potential in dental implantology is undeniable. However, as with any innovation, the field must proceed judiciously, mindful of its constraints, and driven by the imperative for empirical validation.
